# Effect Western Medicines Combined With Nao-Xue-Shu in Patients With Hypertensive Intracerebral Hemorrhage: A Network Meta-Analysis

**DOI:** 10.3389/fphar.2022.892904

**Published:** 2022-06-15

**Authors:** Li Mei, Mu Fengqun, Liu Xiaozhuo, Wang Qing, Fan Mingming, Zuo Zhengyao, Su Dongpo, Han Qian, Chen Tong

**Affiliations:** ^1^ Department of Neurosurgery, North China University of Science and Technology Affiliated Hospital, Tangshan, China; ^2^ Department of Neurology, Gongren Hospital, Tangshan, China

**Keywords:** hypertensive intracerebral hemorrhage, edaravone, nimodipine, nifedipine, Nao-Xue-Shu, network meta-analysis

## Abstract

**Purpose:** To explore the efficacy of nimodipine, nifedipine, and edaravone (EDA) combined with Nao-Xue-Shu in patients with hypertensive intracerebral hemorrhage (HICH) and to determine the best western medicine combined with Nao-Xue-Shu for treating HICH patients using a ranking method.

**Methods:** After a comprehensive search of the China National Knowledge Infrastructure (CNKI), Wanfang Database, VIP information database, Chinese Biomedical Database (CBM), PubMed, Embase, and Cochrane Library database from the database establishment 31 December 2021, data extraction and quality assessment were conducted for the included articles. The primary outcome measure was the effectiveness after treatment. Secondary outcome measures were after-treatment the National Institutes of Health Stroke Scale (NIHSS) scores, hematoma volume, perihematoma edema volume, and inflammatory factor expression levels. Statistical analyses were performed using Stata 16.0 and RevMan 5.3.0 software.

**Results:** We included 19 randomized controlled trials (RCTs) and six non-RCTs. The effective rate after treatment was ranked from the best to the worst as follows: routine cure measure (RCM) + nifedipine + Nao-Xue-Shu, RCM + EDA + Nao-Xue-Shu, RCM + Nao-Xue-Shu, RCM + nimodipine + Nao-Xue-Shu, RCM + EDA, and RCM. The post-treatment NHISS scores from lowest to highest were as follows: RCM + EDA + Nao-Xue-Shu, RCM + nifedipine + Nao-Xue-Shu, RCM + EDA, RCM + nimodipine + Nao-Xue-Shu, RCM + Nao-Xue-Shu, RCM + Nao-Xue-Kang, and RCM. The post-treatment hematoma volume from minimum to maximum was as follows: RCM + EDA + Nao-Xue-Shu, RCM + nimodipine + Nao-Xue-Shu, RCM + nifedipine + Nao-Xue-Shu, RCM + Nao-Xue-Shu, RCM + Nao-Xue-Kang, and RCM. The post-treatment perihematoma edema volume from minimum to maximum was as follows: RCM + EDA + Nao-Xue-Shu, RCM + nifedipine + Nao-Xue-Shu, RCM + nimodipine + Nao-Xue-Shu, RCM + Nao-Xue-Shu, and RCM. For inflammatory factor expression levels after treatment, IL-6 concentration levels after treatment from lowest to highest wasas follows: RCM + Nao-Xue-Shu, RCM + nifedipine + Nao-Xue-Shu, RCM + nimodipine + Nao-Xue-Shu, RCM + EDA + Nao-Xue-Shu, and RCM. TNF-α concentration levels after treatment from lowest to highest was as follow: RCM + nimodipine + Nao-Xue-Shu, RCM + nifedipine + Nao-Xue-Shu, RCM + Nao-Xue-Shu, and RCM.

**Conclusion:** Nao-Xue-Shu combined with nifedipine showed better effectiveness after treatment in HICH patients compared with the other combinations. Nao-Xue-Shu combined with EDA was more effective for improving neurological function and reducing both hematoma and edema volumes around the hematoma compared with the other combinations. However, Nao-Xue-Shu alone or Nao-Xue-Shu combined with nimodipine may be more effective for reducing proinflammatory factor expression.

## Introduction

Spontaneous intracerebral hemorrhage (SICH) is the deadliest, most disabling, and most difficult type of stroke to treat (Tapia-Pérez et al., 2014). Unlike most other stroke types, its morbidity and mortality rates have not decreased over time. It is estimated that over two million people are affected by intracerebral hemorrhage (ICH) worldwide each year. One-third of ICH patients die within 1 month, and a significant number of survivors are left with a permanent disability (Feigin et al., 2009; Steiner et al., 2011). Hypertension was also found to be the most important independent risk factor for patients with ICH, and it is present in about 50% of patients with ICH (Wityk and Caplan, 1992). Middle-aged and elderly people with untreated hypertension or uncontrolled treated hypertension are at risk of ICH, and this SICH caused by hypertension is called hypertensive ICH (HICH) (Woo et al., 2004; Xu et al., 2017).

For HICH patients, early hematoma removal may alleviate ischemia or remove toxic chemicals, thus reducing damage to the neurological tissue (Xi et al., 2006; Vespa et al., 2013). Surgery has the potential to improve neurological recovery after HICH. However, surgical treatment can only partially remove the hematoma, and completely removing the hematoma takes 3–5 days. In HICH patients, the presence of a post-operative edema band surrounding the hematoma and subsequent harm induced by the surgical procedure may limit the effectiveness of its treatment (Teernstra et al., 2003; Thompson et al., 2015). Furthermore, most prospective randomized controlled trials (RCTs) have failed to show that surgical treatment improves the prognosis in these patients (Auer et al., 1989; Batjer et al., 1990; Mendelow et al., 2005). Therefore, there is an urgent need to explore effective treatment options to improve clinical outcomes in patients with HICH.

In addition to active surgical treatment, many researchers have recently investigated the effectiveness of the conservative use of western medicine (such as antihypertensive drugs, anticoagulants, and dehydrant) and Chinese medicine (such as Nao-Xue-Kang and Nao-Xue-Shu) to replace treatment or as palliative treatment for HICH patients. Among them, Nao-Xue-Shu is a typical traditional Chinese medicine.

Nao-Xue-Shu promotes Qi, activates blood, and removes blood stasis, thereby promoting hematoma absorption, reducing brain edema around the hematoma, modulating inflammatory factor to improve the microenvironment, and reducing free radicals. Some researchers have used it alone or in combination with western medicine to treat HICH patients, and explored their effectiveness in reducing the volume of the hematoma and degree of edema around a hematoma in patients with HICH to improve nerve function damage and regulate inflammatory factors (Jiang et al., 2016). Currently, western drugs that have been explored include nimodipine, nifedipine, and edaravone (EDA), all of which had some effectiveness in improving vascular spasm, scavenging free radicals, and alleviating or preventing secondary brain injury after cerebral hemorrhage (Li et al., 2014; Zhang Z, 2017; Li, 2017; Zhang, 2019). However, there is still a lack of direct-comparison evidence between different western pharmaceuticals paired with Nao-Xue-Shu, and it is unclear which western drugs combined with Nao-Xue-Shu are best for patients with HICH. Therefore, the network meta-analysis indirect comparison principle was used to explore the efficacy of nimodipine, nifedipine, and EDA combined with Nao-Xue-Shu in patients with HICH and to analyze which western medicine combined with Nao-Xue-Shu is best for treating HICH patients using a sequencing method.

## Methods

The systematic review and network meta-analysis were performed according to the checklist of the Preferred Reporting Items for Systematic Reviews and Meta-analyses (PRISMA) extension statement for network meta-analysis.

### Inclusion and Exclusion Criteria

The inclusion and exclusion criteria for this study were in accordance with the PICO (P: patient, I: intervention, C: comparison, O: outcome) principle. The inclusion criteria were as follows: patients with a history of hypertension and computed tomography scan-confirmed ICH at the time of the first onset and at least 18 years of age. In the intervention group, patients received Nao-Xue-Shu or Nao-Xue-Shu combined with a western medicine treatment in addition to the routine cure measures (RCM). In the control group, patients RCM alone or RCM combined with a western medicine treatment. Study types included RCTs or non-RCTs that enrolled at least 25 people. The primary outcome indicator was the clinical response rate after treatment (response rate = mostly cured + significant improvement + improvement) on the basis of the National Institutes of Health Stroke Scale (NIHSS) neurological deficit scores, which was classified as follows: 1) mostly cured, where the neurological deficit score decreased by 91–100 percent, and the disability degree was grade 0; 2) significantly improved, where the neurological deficit score decreased by 46–90 percent, and the disability degree was grade 1 to 3; 3) improved, where the neurological deficit score decreased by 18–45 percent; 4) no change, where the neurological deficit score decreased by 17 percent; 5) deterioration, where the neurological deficit score decreased or increased by more than 18 percent; and 6) death. Secondary outcome indicators were the volume of cerebral hematoma and edema after treatment and the concentration levels of interleukin (IL)-6 and tumor necrosis factor (TNF)-α after treatment.

The exclusion criteria were as follows: publications including patients with cerebral hemorrhage caused by rupture of cerebral arteriovenous malformations or trauma; publications including patients with severe heart, lung, liver, kidney, or coagulation dysfunction; single-arm trials; animal trials; and case reports.

### Literature Search

“Intracranial hemorrhage,” “intracerebral hemorrhage,” “brain hemorrhage,” “Naoxueshu,” “Nao-Xue-Shu,” and “Nao Xue Shu” were used as MeSH search terms and keywords. A comprehensive search was conducted using the China National Knowledge Infrastructure (CNKI), Wanfang Database, VIP information database, Chinese Biomedical Database (CBM), PubMed, Embase, and Cochrane Library database from the database establishment 31 December 2021. Relevant references, abstracts of conference papers, ongoing or unpublished trials in World Health Organization clinical registries, and relevant meta-analyses or systematic reviews published in the past 3 years were searched manually and retrieved.

### Data Screening and Quality Evaluation

All the retrieved publications were independently screened by two reviewers. The included RCTs were evaluated using the six aspects of the Cochrane risk of bias tool, as follows: randomization, allocation hiding, blind application, data integrity, selective reporting, and other biases. The included non-RCTs were evaluated using the three aspects of the Newcastle–Ottawa scale, as follows: selectivity, comparability, and results. Any problems or disagreements encountered in the process of screening, including analysis articles and quality assessment, were resolved by two reviewers after consultation or by a third reviewer through consultation.

### Data Extraction

The following data from all the included articles were extracted using Microsoft Excel worksheets: author, publication year, country, study type, age, intervention measures, number of participants in each intervention group, and clinical outcome indicators (primary and secondary outcome indicators). For studies with missing data, the original author was contacted to try to obtain the data.

### Statistical Analysis

The RevMan 5.3 (Cochrane Collaboration, London, United Kingdom) was used for paired meta-analysis. The relative ratio (RR) and 95% confidence interval (CI) were used for dichotomous data. The mean and standard deviation (SD) were used to evaluate the efficacy of different treatment regimens for continuous data. The heterogeneity was assessed using the Cochrane Q test and I^2^ statistic. I^2^<50% was considered to have low heterogeneity and a fixed-effect model was used, while I^2^>50% was considered to have high heterogeneity and a random-effect model was used. A funnel plot was performed when more than 10 articles were included, which was used to evaluated the potential publication bias. Statistical difference was considered when the two-sided *p* value was less than 0.05.

The network meta-analysis was performed using Stata 16.0 software (StataCorp, College Station, TX United States) to analyze the efficacy of different interventions. Before evaluating the direct and indirect evidence, we use the cut-off point method to verify whether there was inconsistency in the Network model. We used surface under the cumulative ranking curves (SUCRA) to rank the interventions for each outcome.

## Results

### Literature Search

**FIGURE 1:**
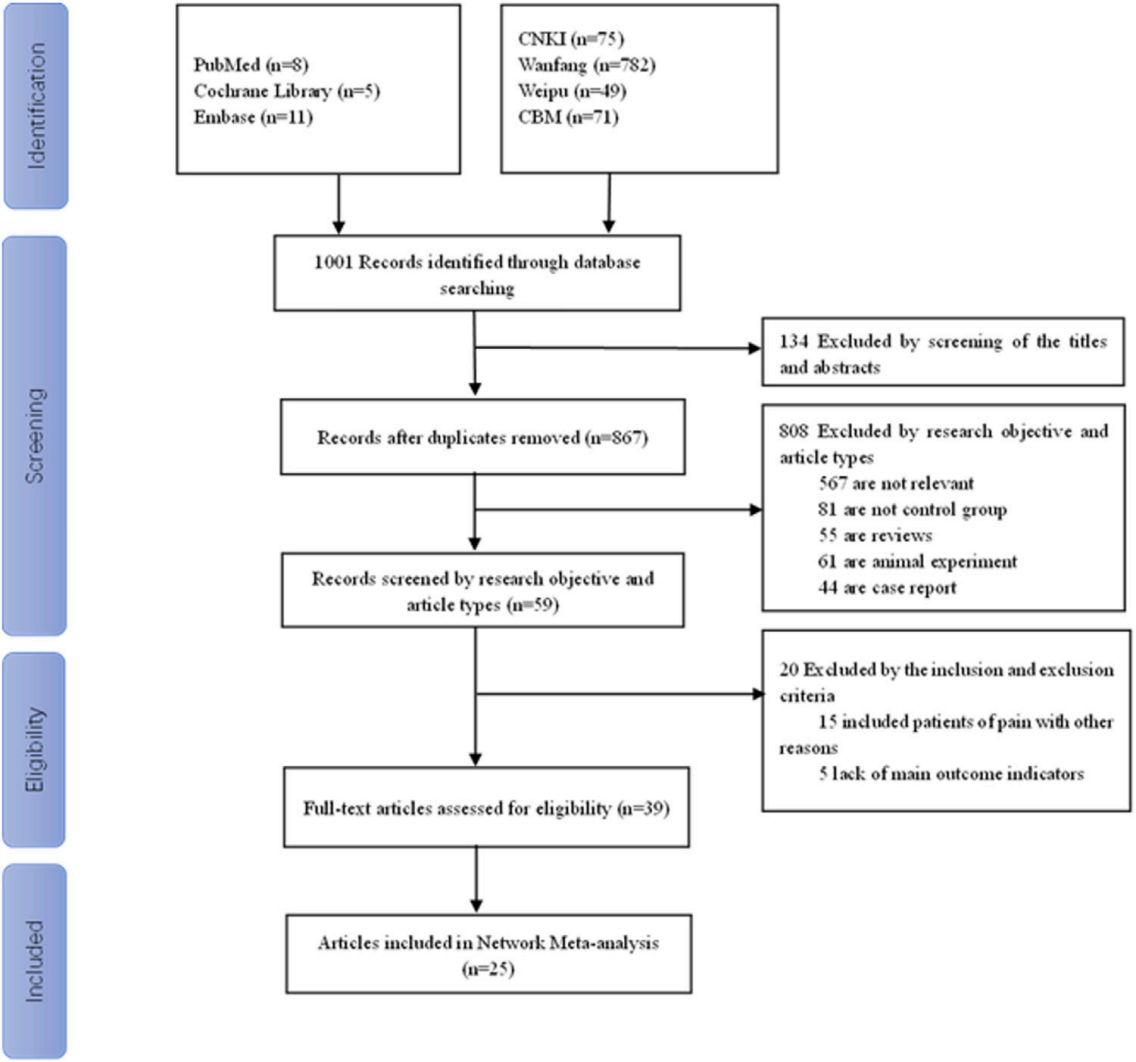
Flow chart of the study selection process.

There were 1,001 articles that were retrieved. Among them, 134 duplicate articles were deleted after reading the titles and abstracts, and 808 articles were deleted on the basis of the research purpose and article type. Additionally, 20 articles were deleted on the basis of the inclusion and exclusion criteria. Finally, 25 articles were included in the network meta-analysis. The flow chart of the study selection process is shown in Figure 1. Nineteen RCT articles (Lu et al., 2004; Zhang and Ji, 2011; Yao, 2012; Li et al., 2014; Miao and Yan, 2014; Wang et al., 2014; Jiang et al., 2016; Wang et al., 2016; Zhang Z, 2017; Zhang T. J, 2017; Li, 2017; Wei and Ma, 2017; Yang et al., 2017; Zhu et al., 2017; Yi and Zeng, 2018; Zhou et al., 2018; Hou et al., 2019; Yang, 2019; Chen and Ma, 2020) and six non-RCT articles (Yang et al., 2015; Guo et al., 2017; Hao et al., 2018; Duan et al., 2019; Wang et al., 2019; Zhang, 2019) were included in the network meta-analysis, with a total sample size of 2,335 patients. Types of included studies, types of interventions, and other details are shown in Supplementary Table S1.

### Quality Evaluation

**FIGURE 2:**
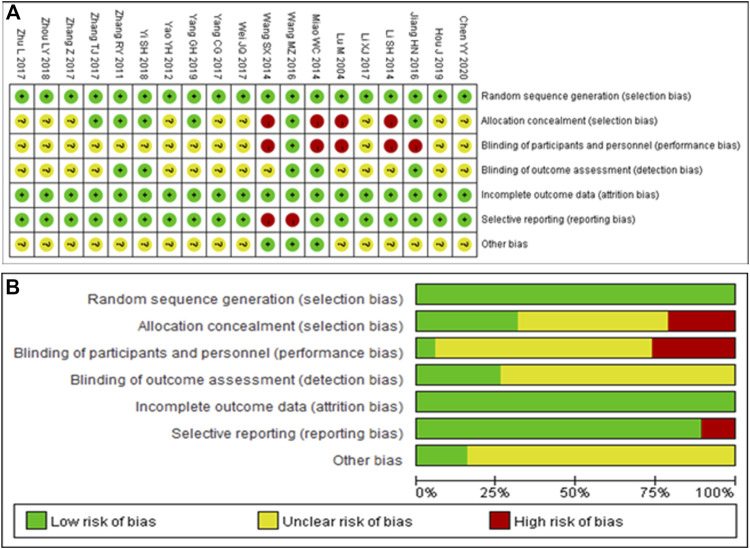
Quality assessment of the included randomized controlled trials. **(A)** Each risk of bias item is presented as the percentages across all included studies. **(B)** Each risk of bias item for each included study. Green indicates a low risk of bias, yellow indicates an unclear risk of bias, and red indicates a high risk of bias.

Nineteen RCTs were included in the analysis, all of which used the correct randomization method and had complete data. Except for Wang et al. (2014) (Wang et al., 2014) and Wang et al. (2016) (Wang et al., 2016), there were no selective reports. However, it is unclear whether implementation of the allocation concealment and blinding was performed correctly in most studies. Thus, the quality of the RCTs included in the analysis was moderate (Figure 2). The Newcastle–Ottawa Scale assessment tool was used for the six non-RCTs, which scored high in selectivity, comparability, and results (Supplementary Table S2), indicating that the included non-RCTs were of high quality.

### Traditional Meta-Analysis and Publication Bias

Using the fixed-effect model, a subgroup analysis of the post-treatment efficiency of the different interventions revealed that there was no heterogeneity between subgroups (I^2^<50%, *p* > 0.1; Supplementary Figure S1). This analysis revealed that, compared with RCM, RCM combined with Nao-Xue-Shu, RCM combined with nimodipine and Nao-Xue-Shu, and RCM combined with nifedipine and Nao-Xue-Shu, RCM combined with EDA and Nao-Xue-Shu had higher post-treatment efficiency. Additionally, RCM combined with EDA and Nao-Xue-Shu also had a higher post-treatment response rate than RCM combined with EDA. Using the random-effect model, subgroup analysis of the NIHSS scores after treatment with different interventions indicated that there was significant heterogeneity between the subgroups (I^2^>50%, *p* < 0.1; Supplementary Figure S2). This analysis showed that compared with RCM, RCM combined with Nao-Xue-Shu, RCM combined with nifedipine and Nao-Xue-Shu, and RCM combined with EDA and Nao-Xue-Shu had a lower NIHSS scores after treatment (the lower the NIHSS scores, the better the patient’s neurological function). However, the NIHSS scores of RCM combined with nimodipine were not lower than that of RCM alone. Additionally, NIHSS scores of RCM combined with EDA and Nao-Xue-Shu were not lower than RCM combined with EDA.

**FIGURE 3:**
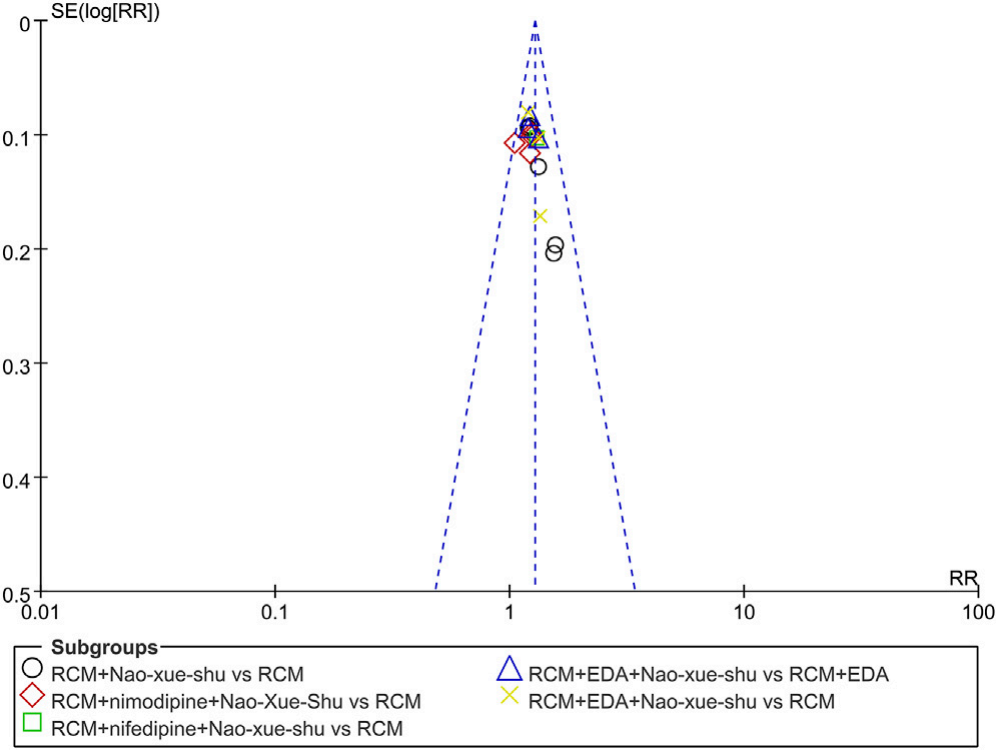
Funnel plots for the detection of publication bias on the post-treatment efficicency.

A funnel plot analysis was performed on the post-treatment efficiency of the two interventions, revealed that no evidence of publication bias was observed for the comparison and the results were statistically robust (Figure 3).

### Network Meta-Analysis

#### Network Diagram of Different Intervention Measures

**FIGURE 4:**
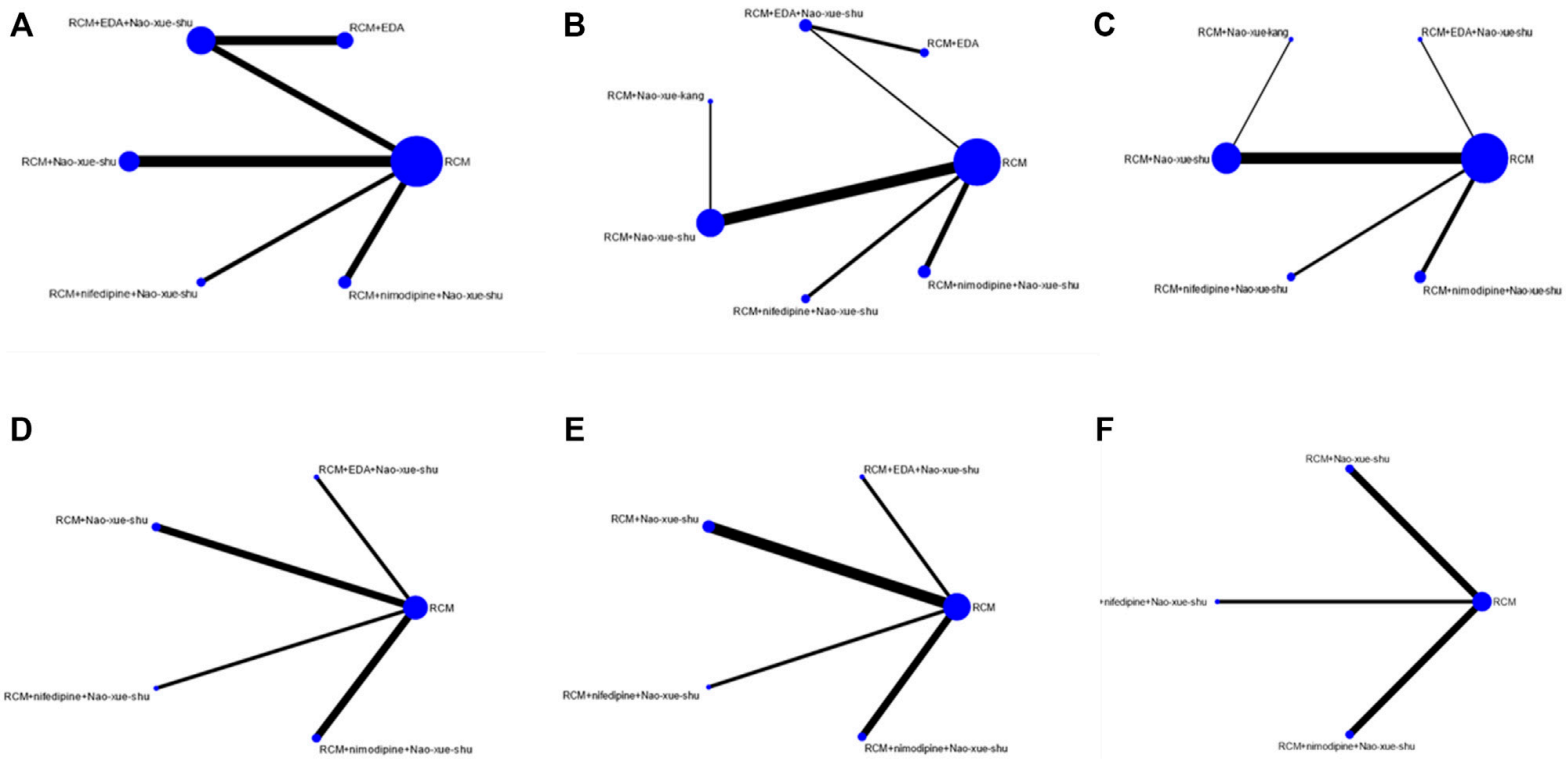
Network charts. Network charts based on the **(A)** effectiveness of different interventions, **(B)** neurological deficit score, **(C)** hematoma volume after treatment, **(D)** edema volume after treatment, **(E)** IL-6 concentration after treatment, and **(F)** TNF-α concentration after treatment. IL, interleukin; TNF, tumor necrosis factor.

A direct comparison is shown if there is a direct line between the two intervention groups, but if there is no line, there is no evidence of a direct comparison. The dot size in the figure represents the sample size, and the line thickness represents the number of studies. RCM was found to be the most frequent control (Figures 4A–F).

#### Inconsistency Test

There was no direct or indirect comparative evidence in the included studies, so no inconsistency test was conducted.

#### Sequence Diagram of Network Meta-Analysis

**FIGURE 5:**
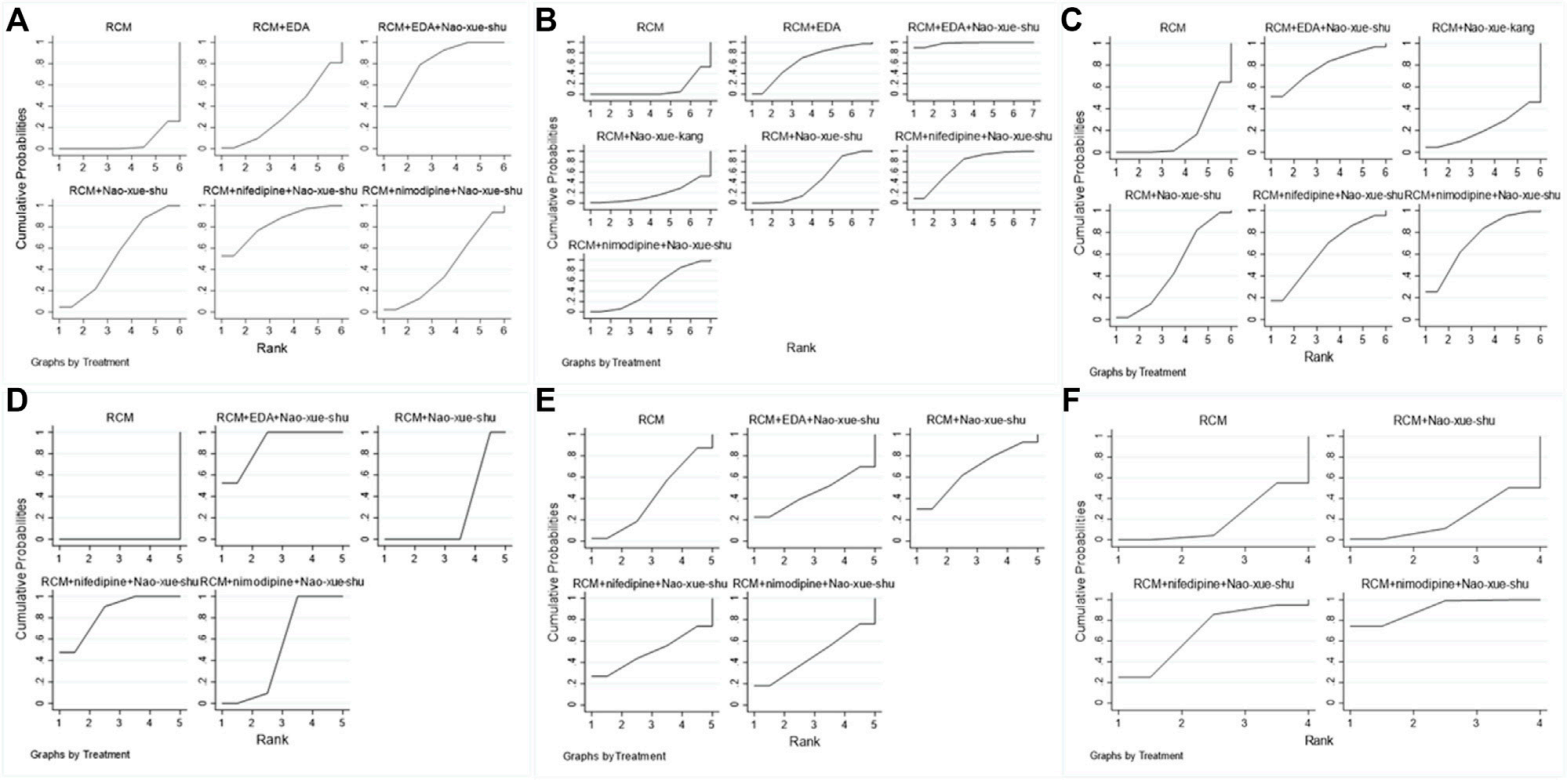
The rank charts. Rank charts based on the **(A)** effectiveness of different interventions, **(B)** neurological deficit score, **(C)** hematoma volume after treatment, **(D)** edema volume after treatment, **(E)** IL-6 concentration after treatment, and **(F)** TNF-α concentration after treatment IL, interleukin; TNF, tumor necrosis factor.

Among the articles included in the analysis, 17 reported post-treatment response rates, involving six different interventions, for which a network meta-analysis was performed (Figure 5A). This analysis revealed that RCM combined with nifedipine and Nao-Xue-Shu, RCM combined with EDA and Nao-Xue-Shu, RCM combined with Nao-Xue-Shu, RCM combined with nimodipine and Nao-Xue-Shu, and RCM combined with EDA had higher post-treatment effective rate than RCM for treating patients with HICH. The order of post-treatment effectiveness from best to worst was as follows: RCM combined nifedipine and Nao-Xue-Shu, RCM combined EDA and Nao-Xue-Shu, RCM combined Nao-Xue-Shu, RCM combined nimodipine and Nao-Xue-Shu, RCM combined EDA, and finally RCM.

There were 15 articles that included the NIHSS scores after treatment, involving seven different interventions. The network meta-analysis (Figure 5B) revealed that RCM combined with EDA and Nao-Xue-Shu, RCM combined with nifedipine and Nao-Xue-Shu, RCM combined with EDA, RCM combined with nimodipine and Nao-Xue-Shu, RCM combined with Nao-Xue-Shu, and RCM combined with Nao-Xue-Kang had lower post-treatment NIHSS scores than RCM for treating patients with HICH. The order of post-treatment NIHSS scores from lowest to highest was as follows: RCM combined with EDA and Nao-Xue-Shu, RCM combined with nifedipine and Nao-Xue-Shu, RCM combined with EDA, RCM combined with nimodipine and Nao-Xue-Shu, RCM combined with Nao-Xue-Shu, RCM combined with Nao-Xue-Kang, and finally RCM.

For the post-treatment hematoma volume, 14 studies involving six different interventions were analyzed in the network meta-analysis (Figure 5C). This analysis revealed that RCM combined with EDA and Nao-Xue-Shu, RCM combined with nimodipine and Nao-Xue-Shu, RCM combined with nifedipine and Nao-Xue-Shu, RCM combined with Nao-Xue-Shu, and RCM combined with Nao-Xue-Kang had a smaller post-treatment hematoma volume than that of RCM for treating patients with HICH. The order of post-treatment hematoma volume from minimum to maximum was as follows: RCM combined with EDA and Nao-Xue-Shu, RCM combined with nimodipine and Nao-Xue-Shu, RCM combined with nifedipine and Nao-Xue-Shu, RCM combined with Nao-Xue-Shu, RCM combined with Nao-Xue-Kang, and finally RCM.

For the post-treatment perihematoma edema volume, six studies involving five different interventions were analyzed using a network meta-analysis (Figure 5D). This analysis revealed that RCM combined with EDA and Nao-Xue-Shu, RCM combined with nifedipine and Nao-Xue-Shu, RCM combined with nimodipine and Nao-Xue-Shu, and RCM combined with Nao-Xue-Shu showed a smaller post-treatment edema volume than that of RCM for treating patients with HICH. The order of post-treatment edema volume from minimum to maximum was as follows: RCM combined with EDA and Nao-Xue-Shu, RCM combined with nifedipine and Nao-Xue-Shu, RCM combined with nimodipine and Nao-Xue-Shu, RCM combined with Nao-Xue-Shu, and finally RCM.

There were seven reports that presented the IL-6 concentration levels after treatment, involving five different interventions. A network meta-analysis was performed on these seven studies (Figure 5E), and it revealed that RCM combined with Nao-Xue-Shu, RCM combined with nifedipine and Nao-Xue-Shu, RCM combined with nimodipine and Nao-Xue-Shu, and RCM combined with EDA and Nao-Xue-Shu showed lower post-treatment IL-6 concentration levels than those of RCM when treating HICH patients. The order of post-treatment IL-6 concentration levels from lowest to highest was as follows: RCM combined with Nao-Xue-Shu, RCM combined with nifedipine and Nao-Xue-Shu, RCM combined with nimodipine and Nao-Xue-Shu, RCM combined with EDA and Nao-Xue-Shu, and finally RCM.

Additionally, there were five reports that presented the TNF-α concentration levels after treatment, involving four different interventions. The network meta-analysis (Figure 5F) revealed that RCM combined with nimodipine and Nao-Xue-Shu and RCM combined with nifedipine and Nao-Xue-Shu had lower post-treatment TNF-α concentration levels than RCM combined with those of Nao-Xue-Shu and RCM when treating HICH patients. The order of post-treatment TNF-α concentration levels from lowest to highest was as follows: RCM combined with nimodipine and Nao-Xue-Shu, RCM combined with nifedipine and Nao-Xue-Shu, RCM combined with Nao-Xue-Shu, and RCM.

## Discussion

HICH is a common neurosurgical disease, which can be life-threatening and also cause a heavy economic burden to patients’ families and to society (Zhang et al., 2014). Although adverse effects associated with HICH are well known, there have been no major advances in treatment regimens to date (Tang et al., 2018). Traditional medicine, especially Chinese medicine, is a complete medical system with thousands of years of application history, and its clinical practice mainly focuses on diagnosis and treatment. Chinese medicine has been shown to play a vital role in treating diseases such as diabetes, cancer, and rheumatoid arthritis (Wang S et al., 2021; Wang Y et al., 2021; Li et al., 2021; Xiang et al., 2021). Therefore, an increasing number of Chinese scholars began to explore the efficacy and safety of Chinese medicine therapy in patients with HICH.

Nao-Xue-Shu is a traditional Chinese patent medicine that is composed of astragalus root, leech, stone calamus, *Achyranthes*, cortex moutan, rhubarb, and Chuanxiong. Based on its beneficial effects of tonifying Qi, activating blood, and removing blood stasis, an increasing number of researchers are exploring the efficacy of this medicine alone or in combination with western medicine to treat HICH patients. However, it is unclear whether this drug is more effective when used alone or in combination with western medicine, and the best western medicine combined with Nao-Xue-Shu to treat HICH patients has not been determined.

Thus, the network meta-analysis indirect comparison principle was used to comprehensively search the existing clinical trials involving HICH treatment with Nao-Xue-Shu alone or in combination with different western medicines. Nineteen RCTs (Lu et al., 2004; Zhang and Ji, 2011; Yao, 2012; Li et al., 2014; Miao and Yan, 2014; Wang et al., 2014; Jiang et al., 2016; Wang et al., 2016; Zhang Z, 2017; Zhang T. J, 2017; Li, 2017; Wei and Ma, 2017; Yang et al., 2017; Zhu et al., 2017; Yi and Zeng, 2018; Zhou et al., 2018; Hou et al., 2019; Yang, 2019; Chen and Ma, 2020) and six non-RCTs (Yang et al., 2015; Guo et al., 2017; Hao et al., 2018; Duan et al., 2019; Wang et al., 2019; Zhang, 2019) were included. The results showed that compared with RCM, treating HICH patients with RCM + nifedipine + Nao-Xue-Shu, RCM + EDA + Nao-Xue-Shu, RCM + Nao-Xue-Shu, RCM + nimodipine + Nao-Xue-Shu, or RCM + EDA showed higher post-treatment effectiveness. HICH patients treated with RCM + EDA + Nao-Xue-Shu, RCM + nifedipine + Nao-Xue-Shu, RCM + EDA, RCM + nimodipine + Nao-Xue-Shu, RCM + Nao-Xue-Shu, or RCM + Nao-Xue-Kang showed lower post-treatment NIHSS scores compared with those of RCM. Additionally, RCM + EDA + Nao-Xue-Shu, RCM + nimodipine + Nao-Xue-Shu, RCM + nifedipine + Nao-Xue-Shu, RCM + Nao-Xue-Shu, or RCM + Nao-Xue-Kang treatment in HICH patients showed a smaller post-treatment hematoma volume compared with that of RCM, while RCM + EDA + Nao-Xue-Shu, RCM + nifedipine + Nao-Xue-Shu, RCM + nimodipine + Nao-Xue-Shu, or RCM + Nao-Xue-Shu showed a smaller post-treatment edema volume compared with RCM. Moreover, RCM + Nao-Xue-Shu, RCM + nifedipine + Nao-Xue-Shu, RCM + nimodipine + Nao-Xue-Shu, or RCM + EDA + Nao-Xue-Shu showed lower post-treatment IL-6 and TNF-α concentration levels compared with RCM.

Combined with the above analysis, Nao-Xue-Shu combined with nimodipine, nifedipine, or EDA had higher post-treatment efficiency and also significantly improved the neurological function compared with that of Nao-Xue-Shu alone. Additionally, the above-mentioned combinations of Chinese and western drugs reduced both the hematoma and edema volumes around hematoma and the release of pro-inflammatory factors compared with Nao-Xue-Shu alone. Among these combinations, Nao-Xue-Shu combined with nifedipine improved treatment effectiveness the most, while Nao-Xue-Shu combined with EDA improved the neurological function and reduced the hematoma and edema volumes around hematoma the most compared with the other groups. Furthermore, treatment with Nao-Xue-Shu alone or Nao-Xue-Shu combined with nimodipine may be more effective in reducing the expression level of pro-inflammatory factors compared with the other groups. Thus, we found that for HICH, various western drugs combined with Nao-Xue-Shu had their own therapeutic advantages. However, it remains unclear which western drugs combined with Nao-Xue-Shu are more suitable for HICH patients.

There are some limitations in this study. First, all studies included in the analysis were research from China, and because the included studies were not from any other countries or ethnic groups, we cannot determine whether Nao-Xue-Shu combined with western medicine can be generalized to other countries and ethnicities. Second, this analysis included non-RCTs and medium-quality RCTs, so high-quality, large-scale, multi-center RCTs are still needed to further verify the efficacy and safety of Nao-Xue-Shu combined with different western drugs to treat HICH patients.

## Conclusion

The results of this network meta-analysis suggest that Nao-Xue-Shu combined with nifedipine showed better effectiveness after treatment in HICH patients compared with the other combinations. Nao-Xue-Shu combined with EDA was more effective for improving neurological function and reducing both hematoma and edema volumes around the hematoma compared with the other combinations. However, Nao-Xue-Shu alone or Nao-Xue-Shu combined with nimodipine may be more effective for reducing proinflammatory factor expression.

## Data Availability

The original contributions presented in the study are included in the article/Supplementary Material, further inquiries can be directed to the corresponding author.
