# Supplementary material for: Effect Western Medicines Combined With Nao-Xue-Shu in Patients With Hypertensive Intracerebral Hemorrhage: A Network Meta-Analysis
Source: Front Pharmacol. 2022 Jun 15;13:892904. doi: 10.3389/fphar.2022.892904 (PMC9240398; doi:10.3389/fphar.2022.892904)
Supplement: Supplementary file 1 [file Table1.DOCX]

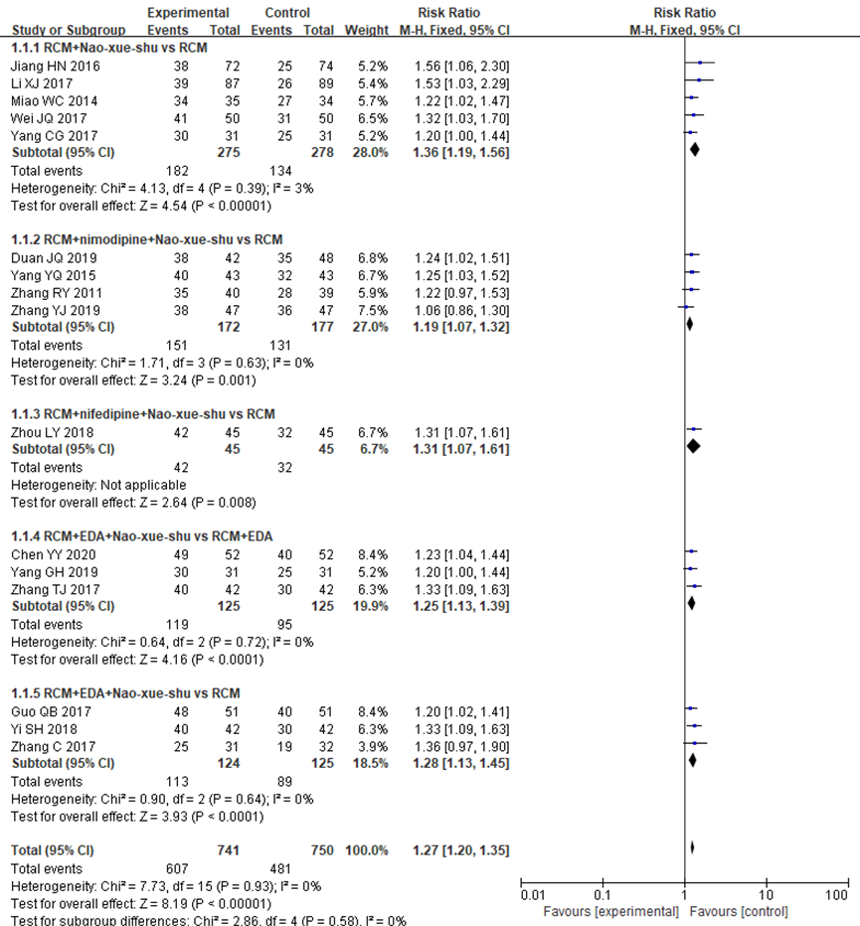


Supplementary figure 1. Subgroup analysis of the effectiveness of different interventions after treatment


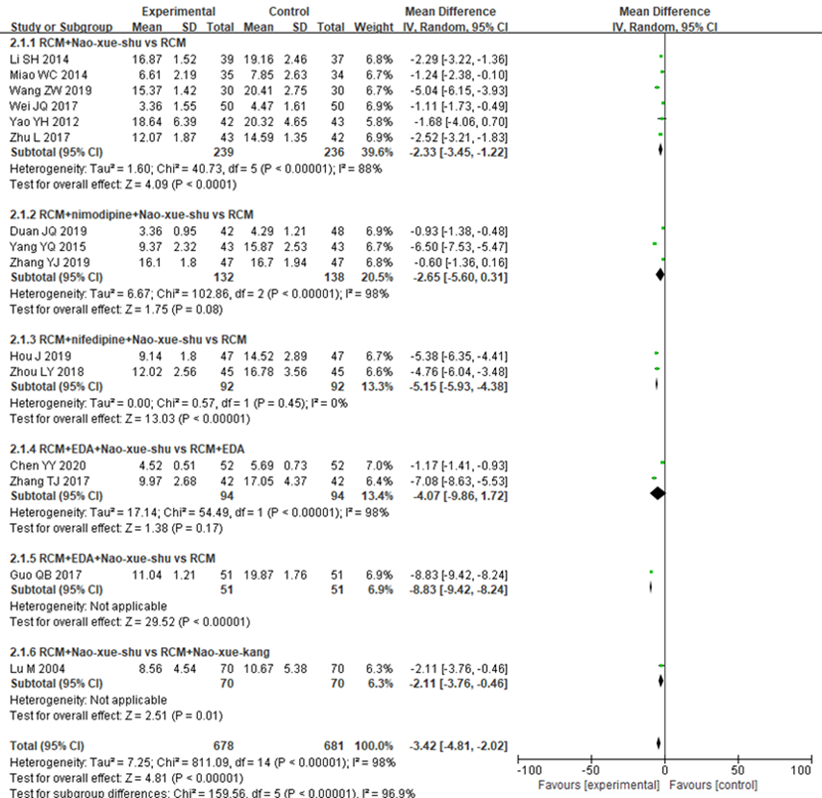


Supplementary figure 2. Subgroup analysis of the NIHSS score after treatment
