# Supplementary material for: Effect Western Medicines Combined With Nao-Xue-Shu in Patients With Hypertensive Intracerebral Hemorrhage: A Network Meta-Analysis
Source: Front Pharmacol. 2022 Jun 15;13:892904. doi: 10.3389/fphar.2022.892904 (PMC9240398; doi:10.3389/fphar.2022.892904)
Supplement: Supplementary file 2 [file Table2.DOCX]

Supplementary Table 1. Overview of the included literature

| Study | Country | Publication year | Study design | Study type | Intervention/Control | Treatment details | Numbers | Treatment cycle |
| --- | --- | --- | --- | --- | --- | --- | --- | --- |
| Zhang Z | China | 2017 | Random | Monocenter | RCM+EDA+Nao-xue-shu/RCM | Intravenous infusion of edaravone; Nao-Xue-Shu oral liquid was given 10ml, three times per day for 14 days. | 31/32 | 2-week |
| Li XJ | China | 2017 | Random | Monocenter | RCM+Nao-xue-shu/RCM | Nao-Xue-Shu oral liquid was given 10ml, three times per day for 14 days. | 87/89 | 2-week |
| Li SH | China | 2014 | Random | Monocenter | RCM+Nao-xue-shu/RCM | Nao-Xue-Shu oral liquid was given 10ml, three times per day for 14 days. | 39/37 | 2-week |
| Zhang YJ | China | 2019 | non-Random | Monocenter | RCM+nimodipine+Nao-xue-shu/RCM | Intravenous infusion of nimodipine, three times per day with  40 mg; Nao-Xue-Shu oral liquid was given 10ml, three times  per day for 14 days. | 47/47 | 4-week |
| Wang SX | China | 2014 | Random | Monocenter | RCM+Nao-xue-shu/RCM | Nao-Xue-Shu oral liquid was given 10ml, three times  per day for 14 days. | 64/58 | 4-week |
| Wei JQ | China | 2017 | Random | Monocenter | RCM+Nao-xue-shu/RCM | Nao-Xue-Shu oral liquid was given 10ml, three times  per day for 14 days. | 50/50 | 4-week |
| Hao Y | China | 2018 | non-Random | Monocenter | RCM+Nao-xue-shu/RCM | Nao-Xue-Shu oral liquid was given 10ml, three times  per day for 10 days. | 41/43 | 10-d |
| Duan JQ | China | 2019 | non-Random | Monocenter | RCM+nimodipine+Nao-xue-shu/RCM | Intravenous infusion of nimodipine, three times per day with  40 mg; Nao-Xue-Shu oral liquid was given 10ml, three times  per day for 14 days. | 42/48 | 4-week |
| Wang ZW | China | 2019 | non-Random | Monocenter | RCM+Nao-xue-shu/RCM | Nao-Xue-Shu oral liquid was given 10ml, three times per day for 14 days. | 30/30 | 4-week |
| Yang YQ | China | 2015 | non-Random | Monocneter | RCM+nimodipine+Nao-xue-shu/RCM | Intravenous infusion of nimodipine, three times per day with  40 mg; Nao-Xue-Shu oral liquid was given 10ml, three times  per day for 14 days. | 43/43 | —— |
| Zhou LY | China | 2018 | Random | Monocenter | RCM+nifedipine+Nao-xue-shu/RCM | Nifedipine orally, twice a day with 10ml; Nao-Xue-Shu oral liquid was given 10ml, three times per day for 14 days. | 45/45 | 3-week |
| Guo QB | China | 2017 | non-Random | Monocenter | RCM+EDA+Nao-xue-shu/RCM | Intravenous infusion of edaravone, twice a day with 30 mg; Nao-Xue-Shu oral liquid was given 10ml, three times per day for 14 days. | 51/51 | 2-week |
| Chen YY | China | 2020 | Random | Monocenter | RCM+EDA+Nao-xue-shu/RCM+EDA | Intravenous infusion of edaravone, twice a day with 30 mg for 14 days; Nao-Xue-Shu oral liquid was given 10ml, three times per day for 14 days. | 52/52 | 4-week |
| Yang GH | China | 2019 | Random | Monocenter | RCM+EDA+Nao-xue-shu/RCM+EDA | Intravenous infusion of edaravone, twice a day with 30 mg; Nao-Xue-Shu oral liquid was given 10ml, three times per day for 14 days. | 40/40 | 2-week |
| Zhang TJ | China | 2017 | Random | Monocenter | RCM+EDA+Nao-xue-shu/RCM | Intravenous infusion of edaravone, twice a day with 30 mg for 14 days; Nao-Xue-Shu oral liquid was given 10ml, three times per day for 28 days. | 42/42 | 4-week |
| Zhu L | China | 2017 | Random | Monocenter | RCM+Nao-xue-shu/RCM | Nao-Xue-Shu oral liquid was given 10ml, three times per day for 1 month. | 43/42 | 1-month |
| Miao WC | China | 2014 | Random | Monocenter | RCM+Nao-xue-shu/RCM | Nao-Xue-Shu oral liquid was given 10ml, three times per day for 1 month. | 35/34 | 1-month |
| Lu M | China | 2004 | Random | Monocenter | RCM+Nao-xue-shu /RCM+Nao-xue-kang | Nao-Xue-Shu oral liquid was given 10ml, three times per day for 1 month; Nao-Xue-Kang oral liquid was given 10ml, three times per day for 1 month. | 70/70 | 1-month |
| Hou J | China | 2019 | Random | Monocenter | RCM+nifedipine+Nao-xue-shu/RCM | Nifedipine orally, twice a day with 10ml; Nao-Xue-Shu oral liquid was given 10ml, three times per day for 28 days. | 47/47 | 4-week |
| Yang CG | China | 2017 | Random | Monocenter | RCM+Nao-xue-shu/RCM | Nao-Xue-Shu oral liquid was given 10ml, three times per day for 2 weeks. | 31/31 | 3-month |
| Zhang RY | China | 2011 | Random | Monocenter | RCM+EDA+Nao-xue-shu/ RCM+EDA | Intravenous infusion of edaravone, twice a day with 30 mg for 14 days; Nao-Xue-Shu oral liquid was given 10ml, three times per day for 14 days. | 40/39 | 2-week |
| Yi SH | China | 2018 | Random | Monocenter | RCM+EDA+Nao-xue-shu/  RCM | Intravenous infusion of edaravone, twice a day with 30 mg for 28 days; Nao-Xue-Shu oral liquid was given 10ml, three times per day for 28 days. | 42/42 | 28-week |
| Wang MZ | China | 2016 | Random | Monocenter | RCM+Nao-xue-shu/RCM | Nao-Xue-Shu oral liquid was given 10ml, three times per day for 2 weeks. | 41/39 | 2-week |
| Yao YH | China | 2012 | Random | Monocenter | RCM+Nao-xue-shu/RCM | Nao-Xue-Shu oral liquid was given 10ml, three times per day  for 2 weeks. | 43/42 | 2-week |
| Jiang HN | China | 2016 | Random | Multicenter | RCM+Nao-xue-shu/RCM | Nao-Xue-Shu oral liquid was given 10ml, three times per day for 2 weeks. | 74/72 | 2-week |

RCM: routine cure medicine; EDV: edaravone

Supplementary Table 2. Quality assessment of the non-RCT studies

| Study | Selection | Comparability | Outcome | Total Score |
| --- | --- | --- | --- | --- |
| Zhang YJ | 4 | 2 | 2 | 8 |
| Hao Y | 4 | 2 | 2 | 8 |
| Duan JQ | 4 | 2 | 3 | 9 |
| Wang ZW | 4 | 1 | 3 | 8 |
| Yang YQ | 4 | 2 | 2 | 8 |
| Guo QB | 4 | 2 | 3 | 9 |

A score of 5 or less indicates a high risk of bias
